# Plasma glucocorticogenic activity, race/ethnicity and alcohol intake among San Francisco Bay Area women

**DOI:** 10.1371/journal.pone.0233904

**Published:** 2020-06-01

**Authors:** Phum Tachachartvanich, Sylvia S. Sanchez, Scarlett L. Gomez, Esther M. John, Martyn T. Smith, Laura Fejerman

**Affiliations:** 1 Division of Environmental Health Sciences, School of Public Health, University of California Berkeley, Berkeley, CA, United States of America; 2 Cancer Prevention Institute of California, Fremont, CA, United States of America; 3 Department of Epidemiology and Biostatistics, Helen Diller Family Comprehensive Cancer Center, University of California San Francisco, San Francisco, CA, United States of America; 4 Division of Oncology and Stanford Cancer Institute, Department of Medicine, Stanford University School of Medicine, Stanford, CA, United States of America; 5 Division of General Internal Medicine and Institute of Human Genetics, Department of Medicine, Helen Diller Family Comprehensive Cancer Center, University of California San Francisco, San Francisco, CA, United States of America; Graduate School of Public Health and Health Policy, City University of New York, UNITED STATES

## Abstract

Racial and ethnic minorities are at higher risk for a variety of diseases. While sociodemographic and lifestyle factors contribute to racial/ethnic health disparities, the biological processes underlying these associations remain poorly understood. Stress and its biological consequences through the glucocorticoid receptor (GR) have been hypothesized to mediate adverse disease outcomes. In fasting morning samples of 503 control women from the San Francisco Bay Area Breast Cancer Study, we used a sensitive Chemical-Activated LUciferase gene eXpression (CALUX) assay to examine the association of sociodemographic and lifestyle factors with plasma glucocorticogenic (G) activity in three racial/ethnic groups. The G activity is a sensitive measure that reflects biological activity of total plasma glucocorticoids including cortisol and glucocorticoid-like compounds. Associations between G activity and sociodemographic and lifestyle factors were examined using multivariable linear regression models. Latina and non-Latina Black (NLB) women had 9% (P = 0.053) and 14% (P = 0.008) lower morning G activity than non-Latina White (NLW) women, respectively. Additionally, we replicated a previously reported association between G activity and alcohol intake (women who drank >10gms had 19% higher G activity than non-drinkers, P = 0.004) in Latina and NLB women. Further research should assess the association between G activity and health outcomes in a prospective cohort so as to characterize the relationship between total plasma G activity in pre-disease state and disease outcomes across different racial/ethnic populations.

## Introduction

Racial and ethnic minority populations in the U.S. are at higher risk for a variety of diseases including high blood pressure [1], cardiovascular disease [2], and cancers [3, 4] and tend to have worse outcomes than non-Latino Whites (NLWs) [5, 6]. As racial and ethnic minorities generally have lower levels of socioeconomic status (SES) and have higher levels of psychosocial stress than NLWs [7, 8], stress may be a possible pathway by which minorities incur greater disease burden. Stress has been associated with increased risk of cardiovascular disease, high blood pressure, stroke, type 2 diabetes mellitus and cancer [9–12]. One mechanistic explanation for stress influencing health disparities is through stress-related alterations in biological response systems. The effects of stress are mediated by the stress hormone cortisol. In response to stressors, the hypothalamus releases corticotropin-releasing hormone (CRH), which binds to CRH receptors on the pituitary gland. As a result of the CRH binding, adrenocorticotropic hormone (ACTH) is released [13] and interacts with its receptor located on the adrenal cortex and further stimulates adrenal release of stress hormone cortisol into the circulation [14]. Cortisol, the most abundant endogenous glucocorticoid, plays a prominent role in maintaining homeostasis in response to metabolism changes and stressful perturbations [14]. Previous studies have reported variation in diurnal cortisol levels by race/ethnicity category [15–17], suggesting that different race/ethnicity categories may have different amount of stressor exposure during the day.

Apart from race/ethnicity, lifestyle factors such as alcohol consumption have been reported to influence plasma cortisol levels. Specifically, high alcohol consumption stimulates the release of ACTH and glucocorticoids [18, 19]. Plasma cortisol levels were significantly increased in healthy subjects administrated a high dose of alcohol [20]. Similar findings were observed in animal studies, where an alcohol exposure in rats increased plasma levels of ACTH and glucocorticoids [21].

The biological and physiological actions of glucocorticoids are exerted through the glucocorticoid receptor (GR), which belongs to the classical nuclear receptor superfamily of ligand-dependent transcription factors. Upon glucocorticoid binding, the cortisol-GR complex initiates the transcription of target genes in response to stressors. Interestingly, apart from endogenous glucocorticoids, exogenous chemicals or environmental stressors, like endocrine disruptors that alter the glucocorticogenic (G) signaling pathway, have been found to be associated with adverse health outcomes. For example, bisphenol A (BPA), which is ubiquitously found in polycarbonate plastics and epoxy resins, has been shown to bind GR *in vitro* and *in silico* with similar binding interaction to cortisol and dexamethasone, suggesting an agonistic effect on GR [22]. Furthermore, epidemiological and *in vivo* studies revealed that BPA exposure is associated with anxiety and depression [23, 24]. Voisin *et al*., found that a cholesterol metabolite, 6-oxo-cholestan-3β,5α-diol (OCDO), induces breast cancer proliferation through GR activation and the proliferative effect of OCDO is completely attenuated by a potent GR inhibitor [25]. Collectively, these studies not only emphasize the important role of glucocorticoids in disease pathologies, but also provide evidence that both endogenous and exogenous chemicals that act on the GR have an impact on health. We hypothesized that plasma G activity in racial/ethnic minority groups would differ from G activity in NLWs, which could partly explain the wide spectrum of health disparities in different population groups.

Many studies have investigated the association between stress and adverse health outcomes by measuring cortisol in biological specimens like saliva [26], hair follicles [27], and plasma [28]. These methods lack information regarding the biological activity and summated effects of glucocorticoids and G compounds present in biological specimens. As a result, novel methods that simultaneously capture both endogenous and exogenous chemicals modulating the GR signaling pathway will provide a more complete assessment than measuring cortisol levels alone.

Chemical-Activated LUciferase gene eXpression (CALUX) assays have been widely utilized in epidemiologic research to provide an insight into the biological response to chemical exposure in humans [29, 30]. Luciferase assays measure the summated biological activity of all agonists and antagonists on hormone receptors [30, 31].

In a previous study of foreign-born and U.S.-born Mexican women (N = 90), we found that plasma G activity was associated with alcohol intake [32]. Herein, we aimed to validate/confirm this association and extended the assessment of G activity to include multiple racial/ethnic groups. The summated plasma G activity, which captures all endogenous and exogenous G compounds, was measured in fasting morning samples of women who participated as controls in the San Francisco Bay Area Breast Cancer Study (SFBCS), namely NLW, non-Latina Black (NLB) and Latina women. We examined the association of sociodemographic and lifestyle factors with plasma G activity in these three racial/ethnic groups.

## Materials and methods

### Sample collection and procedure

Participants in the present study were selected from the control group of the SFBCS, which is a multiethnic population-based case-control study of breast cancer as described elsewhere [33]. The SFBCS controls living in San Francisco, San Mateo, Alameda, Contra Costa or Santa Clara counties were identified by random-digit dialing method between 1995 and 2002. Trained professional interviewers administered structured questionnaires in English or Spanish at a home visit that asked about sociodemographic background, medical history, family history of cancer, and lifestyle factors [33]. Dietary intake during the previous calendar year was assessed using the Block Food Frequency Questionnaire. A short questionnaire was administered at the time of blood draw (around 7–11 AM) in order to update some important variables (i.e. menopausal status and alcohol intake). The proportion of Indigenous American (IA) genetic ancestry estimates for the Latina women was determined as described previously [34]. Briefly, global individual IA ancestry was estimated as the average locus-specific ancestry across 59,211 loci for each individual. Locus-specific ancestry estimates were obtained with the HAPMIX software based on a three-way admixture model and included European, African and IA pseudo-ancestral haplotypes as references [34]. In the present study, plasma G activity was measured from 503 women, including 74 NLW, 100 NLB, and 329 Latina women. The age range of the study participants was between 35 to 79 years. The individual-level data are available as a supplementary S1 Dataset. Participants who had missing variable information were removed from the analyses (N = 11).

### Plasma glucocorticogenic activity measurement

To measure plasma G activity, we used a GR mediated CALUX assay, which allowed us to capture total effects of both endogenous and exogenous G compounds. We used procedures as described in the previous study with minor modifications [32]. In short, the MDA-kb2 cell line, a human triple negative breast cancer cell line stably transfected with the murine mammalian tumor virus (MMTV) luciferase neo reporter gene construct, was directly purchased from the American Type Culture Collection (ATCC, catalog no. CRL-2713). These cells highly express both endogenous androgen receptor and GR, which stimulate the MMTV promoter. To measure G activity in plasma, we used a potent androgen receptor inhibitor, hydroxyflutamide (Sigma-Aldrich, St. Louis, USA). MDA-kb2 cells were routinely maintained in Leibovitz’s-15 (L-15) medium (Gibco, Grand Island, NY, USA) supplemented with 10% fetal bovine serum (FBS) (Corning, Bedford, MA, USA) at 37°C in a humidified incubator with no CO_2_. Cell culture media were changed 3 times per week. All cell passage numbers used in this study were less than 10 passages. External sources of total steroids were removed by maintaining the cells with L-15 medium supplemented with 10% charcoal-dextran stripped FBS for 1 week prior to the luciferase assay. The cells were then seeded at a density of 2.7 × 10^4^ cells/well in white, 96-well microtiter plates (Thermo Scientific, Grand Island, NY, USA) and incubated at 37°C for 24 hours. After this initial incubation period, the 80 μL of fasting morning plasma used per female sample was diluted in L-15 medium containing 10% charcoal-dextran stripped FBS and then added in triplicate directly onto cells, in the presence of 5 × 10^−7^ mol/L hydroxyflutamide. After a second incubation of 24 hours, the cells were lysed with 1× passive lysis buffer (Promega, Madison, WI, USA) and the microplate was read using a luminometer (Berthold Technologies, Oak Ridge, TN, USA). Readings from each well were reported in relative light units (RLUs) with higher RLU values indicating greater plasma G activity. The RLUs from triplicate wells were averaged to achieve one measurement of G activity per individual. The overall intra-assay and inter-assay coefficients of variation of this assay were 7–12%. The minimum detection limit for cortisol was 4.4 nmol/L.

### Ethical statement

The study was approved by the Institutional Review Boards at the University of California, San Francisco and the Cancer Prevention Institute of California, and all methods were performed in accordance with the relevant guidelines and regulations. Study participants provided written informed consent.

### Statistical analysis

Differences in means and proportions for analyzed variables between racial/ethnic categories were assessed by two-tailed *t*-test and Fisher’s exact test, respectively. Averaged RLUs from the luciferase assay were natural log (ln) transformed in order to approximate normal distribution. Plate adjusted values were obtained by first estimating average plate effects using linear regression and then subtracting the average plate effect from each individual value. A linear regression analysis was used to analyze the relationship between ln-transformed and plate adjusted G activity as the outcome, with race/ethnicity category, sociodemographic, and lifestyle factors as predictors. Percent change in RLUs per unit change of predictor variables was calculated using the formula [e^β^-1]*100. The cut off for statistical significance was set at a p-value of 0.05 or less. Multivariable regression models included race/ethnicity category (Latinas, NLWs, NLBs), age at blood draw (categorical, <55; 55–65; >65 years), height (continuous, cm), body mass index at interview (BMI: <25 kg/m^2^; 25 to <30 kg/m^2^; ≥30 kg/m^2^), neighborhood socioeconomic status (SES, categorical with values 1 to 5, 1 = lowest SES, 5 = highest SES; estimated using a composite index that included income, education, poverty, unemployment, occupation and housing value, based on 2000 Census block group data [35]) and self-reported alcohol intake at interview (categorical, None; <10 gms daily; 10 or >gms daily) as predictors, and plasma G activity (continuous, ln-transformed) as the outcome variable. These predictor variables included in the models were selected based on previous literature supporting their influences on G activity [32, 36–39]. We also conducted three additional analyses stratifying by racial/ethnic category. A subset of Latina women (n = 279) was used in a separate analysis of Indigenous American (IA) ancestry, sociodemographic, and lifestyle factors. The proportion of IA ancestry was used as a continuous variable ranging from 0 to 100%. The multivariable model for the ancestry analysis included IA ancestry, age at blood draw, height, BMI, neighborhood SES, alcohol consumption and nativity (foreign-born vs. U.S.-born). In addition, subsets of NLW (n = 72) and NLB (n = 99) women were used in the analysis of sociodemographic and lifestyle factors. The multivariable model for the sociodemographic and lifestyle factors analysis included age at blood draw, height, BMI, neighborhood SES, and alcohol consumption. All analyses were performed using Stata software.

## Results

### Baseline characteristics

In the present study, we investigated if individuals from different racial/ethnic groups (NLWs, NLBs, and Latinas) had different plasma G activity as measured by GR mediated luciferase reporter gene assay. Furthermore, we evaluated if plasma G activity was associated with other factors such as age, alcohol intake, BMI, and SES. Demographic characteristics by racial/ethnic category are presented in Table 1. Overall, there was a statistically significant difference in the level of GR RLUs between racial/ethnic categories. G activity was highest among NLW women (mean RLUs of 48845), followed by NLBs (mean RLUs of 43561), and lowest among Latina women (mean RLUs of 42776). Other factors such as age at blood draw, height, place of birth, alcohol intake, BMI, and SES were also significantly different between groups. For example, Latina women were younger based on mean age at blood draw (61 yrs) compared to NLB (62 yrs) and NLW (67 yrs) women. The majority (66%) of the Latina women were foreign-born, while 92% and 97% of the NLW and NLB women were U.S.-born, respectively.

**Table 1:** Demographic characteristics among non-Latina Whites, non-Latina Blacks, and Latinas in the San Francisco Bay Area Breast Cancer Study 1996–2002.

Characteristics	Latinas			NLWs			NLBs			P-value
	N	Mean/%	Sd.	N	Mean/%	Sd.	N	Mean/%	Sd.	
Glucocorticogenic activity in RLUs (plate adjusted) ^a^	329	42776	15182	74	48845	17157	100	43561	15555	0.0056
Age (yrs) ^a^	329	61.49	9.5	74	66.8	10.5	100	61.66	9.9	0.0001
African ancestry proportion ^a^	285	0.08	0.07							
European ancestry proportion ^a^	285	0.51	0.15							
IA ancestry proportion ^a^	285	0.41	0.15							
Height, cm ^a^	324	155.47	6.94	73	161.14	7.34	100	163.31	6.25	<0.0001
Age (yrs) ^b^										
<55	94	28.5		14	18.9		31	31		0.001
55–65	117	35.6		13	17.6		31	31		
>65	118	35.9		47	63.5		38	38		
Place of birth ^b^										
US-born	112	34		68	92		97	97		<0.0001
Foreign-born	217	66		6	8		3	3		
Alcohol Intake per day (gms) ^b^										
None	235	71.4		34	46		76	76		<0.0001
<10	82	24.9		20	27		15	15		
≥10	12	3.7		20	27		9	9		
Socioeconomic status (SES) ^b^										
1 (low SES)	17	5.3		3	4.1		23	23		<0.0001
2	77	23.6		6	8.2		30	30		
3	91	27.9		12	16.4		25	25		
4	80	24.5		21	28.8		11	11		
5 (high SES)	61	18.7		31	42.5		10	10		
BMI (kg/m^2^) ^b^										
<25	42	13		22	30.1		16	16		<0.0001
25 to <30	122	37.6		30	41.1		24	24		
≥30	160	49.4		21	28.8		60	60		
Menopausal status ^b^										
Pre-menopausal	28	9.3		8	11.3		6	6.5		0.56
Post-menopausal	274	90.7		63	88.7		86	93.5		

NLWs, non-Latina Whites; NLBs, non-Latina Blacks; IA, Indigenous American; RLUs, relative light units reported by bioassays.

^a^P-values were calculated using *t*-test.

^b^P-values were calculated using Fisher’s exact test.

### Plasma glucocorticogenic activity

Plasma G activity was obtained for 503 participants. In the univariable analysis, we found NLB and Latina women had 11% (P = 0.018) and 13% (P = 0.001) lower plasma G activity compared to NLWs, respectively. In the multivariable analysis, we observed a significantly lower plasma G activity among Latinas (9%, P = 0.053) and NLBs (14%, P = 0.008) than NLWs (Table 2). We did not observe a linear trend between increasing age and plasma G activity. However, when compared to women who were younger than 55 years, women who were between 55 to 65 years of age had lower G activity (21%, p = 0.016), but the association was not significant in women who were older than 65 years. We observed a positive association between G activity and height. Moreover, women who reported drinking more than 10 gms of alcohol daily had 19% (P = 0.004) higher plasma G activity compared to non-drinkers. The association between G activity and alcohol intake was not observed in women who drank less than 10 gms (Table 2).

**Table 2:** Association of sociodemographic and lifestyle factors with plasma glucocorticogenic activity among non-Latina Whites, non-Latina Blacks, and Latinas (N = 492) in the San Francisco Bay Area Breast Cancer Study 1996–2002.

Characteristics	Coefficient (95% CI)
Age (yrs)	
<55	Ref.
55–65	-0.09 (-0.17, -0.02)
>65	-0.03 (-0.10, 0.05)
Race/ethnicity	
NLW	Ref.
NLB	-0.15 (-0.26, -0.04)
Latina	-0.09 (-0.19, -0.00)
Height, per 10 cm	0.05 (0.00, 0.09)
BMI (kg/m^2^)	
<25	Ref.
25 to <30	0.01 (-0.07, 0.10)
≥30	0.04 (-0.05, 0.12)
Socioeconomic status (SES)	
1 (low SES)	Ref.
2	-0.04 (-0.16, 0.08)
3	-0.05 (-0.17, 0.07)
4	-0.11 (-0.23, 0.01)
5 (high SES)	-0.11 (-0.24, 0.01)
Alcohol intake per day (gms)	
None	Ref.
<10	0.01 (-0.06, 0.09)
≥10	0.17 (0.07, 0.29)

CI, confidence interval; NLW, non-Latina White; NLB, non-Latina Black.

### Subgroup analyses

To further investigate the association between plasma G activity, IA ancestry and other factors, Latina women who had available ancestry information were included in a subgroup analysis. We found plasma G activity was inversely associated with IA ancestry. This inverse association was statistically significant in the univariable analysis (25% change in plasma G activity, P = 0.026), but not in the multivariable analysis (22% change in plasma G activity, P = 0.076) (Table 3). When compared to women who were younger than 55 years, women who were between 55 to 65 years had lower G activity (11%, P = 0.015). The association between plasma G activity and alcohol intake was observed only in women who drank more than 10 gms, with higher G activity in this group compared to nondrinkers (27%, P = 0.021). No other variables in the model were statistically significantly associated with G activity.

**Table 3:** Association of sociodemographic and lifestyle factors with plasma glucocorticogenic activity in Latinas (N = 279) in the San Francisco Bay Area Breast Cancer Study 1996–2002.

Characteristics	Coefficient (95% CI)
Indigenous American ancestry	-0.25 (-0.53, 0.03)
Age (yrs)	
<55	Ref.
55–65	-0.12 (-0.23, -0.02)
>65	-0.03 (-0.13, 0.07)
Height, per 10 cm	0.06 (0.00, 0.13)
BMI (kg/m^2^)	
<25	Ref.
25 to <30	0.02 (-0.11, 0.15)
≥30	0.04 (-0.08, 0.17)
Socioeconomic status (SES)	
1 (low SES)	Ref.
2	-0.06 (-0.25, 0.12)
3	-0.08 (-0.27, 0.10)
4	-0.13 (-0.32, 0.05)
5 (high SES)	-0.14 (-0.33, 0.06)
Alcohol intake per day (gms)	
None	Ref.
<10	-0.00 (-0.10, 0.09)
≥10	0.24 (0.03, 0.44)
Foreign-born	
Yes	-0.06 (-0.15, 0.03)

CI, confidence interval.

NLW and NLB women were included in a separate subset analysis to determine the association between plasma G activity and other factors. We observed a suggestive difference in plasma G activity in NLB women who drank more than 10 gms compared to non-drinkers (23% change in G activity, P = 0.09) (S1 Table). However, this association was not observed in NLW women (S2 Table).

## Discussion

The results of the present study suggest that plasma G activity varies by racial/ethnic categories in the U.S., with lower activity among NLB and Latina women relative to NLW women. In addition, we replicated a previously reported positive association between G activity and alcohol intake [32] and found that among Latina women, those with higher IA ancestry had a lower average level of plasma G activity.

It has been suggested that stress-related disturbance in the hypothalamic-pituitary-adrenal (HPA) axis, especially cortisol regulation, may influence adverse health outcomes. Evidence indicates flatter diurnal cortisol slopes (e.g., due to lower morning and/or elevated evening cortisol levels) among people having stressful life events, difficulty in personal relationships, and trauma [40–42]. Furthermore, flatter diurnal cortisol slopes have also been implicated in the etiology of a variety of disease outcomes such as breast cancer mortality and type 2 diabetes mellitus [43, 44]. Sephton *et al*. found that cortisol slopes can be used as a predictor for breast cancer survival [44]. The study showed that breast cancer patients with lower survival had flatter diurnal cortisol slopes which indicates a lack of normal cortisol rhythms [44]. Although NLB and Latina women have a lower incidence of breast cancer compared to NLW women, they tend to have a higher risk of mortality from the disease [45, 46]. The significantly lower plasma G activity observed among NLB and Latina women relative to NLWs in this study could be explained by a number of reasons. Cortisol, one of the stress biomarkers, is the most abundant endogenous glucocorticoid in the body and has the highest potency over GR [47]. One explanation is that NLB and Latina women may have a blunted cortisol response, which could partially explain observed disparities in disease outcomes. Another explanation could be the exposure to glucocorticoid-like compounds which have been shown to modulate G activity in human studies [23, 25]. To better understand the observed associations of lower G activity in the minority groups, future research should establish experimentally the relationship between plasma G activity, which is sensitive and cost effective, and daytime trajectory of cortisol.

Studies have shown that sociodemographic and lifestyle factors like SES and alcohol consumption are associated with cortisol levels [48]. We found that plasma G activity was associated with alcohol consumption. Women who reported drinking more than 10 gms of alcohol daily had higher plasma G activity compared to non-drinkers, which is in agreement with previous findings [38, 49]. In a study of men and women, alcoholics had about three times greater hair cortisol levels than abstinent alcoholics or non-alcoholics [49]. Abstinent and non-alcoholics had similar hair cortisol levels, indicating that cortisol levels reduce to baseline levels after extended alcohol cessation [49]. Thayer *et al*., reported that healthy men in the highest tertile of self-reported alcohol intake had greater urinary cortisol levels compared to men in the lower tertiles of alcohol intake [38]. Furthermore, these findings support previous studies on the HPA axis dysregulation in heavy drinkers and suggest impaired negative-feedback mechanism of the HPA axis in heavy drinkers [50, 51]. Another mechanism that explains the observed association between alcohol consumption and G activity is the activation of the GR. Inhibition of GR function reduces motives for drinking alcohol, which suggests that higher G activity might lead to higher motivation for alcohol intake [52].

We observed an inverse association between plasma G activity and IA ancestry among Latina women, which may be explained by a blunted cortisol response in Latina women with IA ancestry. In addition, environmental factors such as exposure to exogenous compounds or dietary constituents may also influence the observed association [53]. Dietary constituents such as genistein and daidzein, two phytochemicals which are widely found in beans, directly suppress adrenocortical steroidogenesis through the enzymatic inhibition of 3β-hydroxysteroid dehydrogenase and cytochrome P-450 21-hydroxylase and in turn decrease cortisol production [54]. Moreover, genistein at biological relevant concentrations found in humans [55], has also been shown to decrease plasma ACTH and corticosterone (the most abundant glucocorticoid in rodents) secretion in animal studies [56, 57]. Another explanation could be a genetic difference by ancestral component in lipoprotein and cholesterol metabolism, as some cholesterol metabolites have been shown to affect the GR signaling pathway [25, 58, 59]. Furthermore, a genetic difference in glucocorticoid metabolizing enzymes such as *HSD11B1* and *HSD11B2* could play a prominent role in determining the glucocorticoid bioavailability in circulation which could partly influence plasma G activity. However, these genetic hypotheses warrant further investigation with a larger sample size for adequate statistical power.

There were some limitations that are worth noting. Our study does not account for measures of endogenous cortisol levels, which would have allowed us to calculate the proportion of plasma G activity that might result from differences in the level of exogenous compounds versus endogenous cortisol levels. However, we were able to measure summated G activity which reflects biological activity of glucocorticoid-like compounds of endogenous and exogenous origins, therefore providing a comprehensive representation of total G exposure. Another limitation is that our study had a single time point measurement of plasma G activity and therefore we did not know diurnal cortisol rhythms for each individual. However, the measure of morning glucocorticoid levels has been shown to be associated with chronic stress [60, 61], which substantiates the importance of morning glucocorticoid levels even though it was derived from a single time point. Moreover, we lacked information on the profile of other hormones in the HPA axis that would have allowed us to gain more insight into the whole HPA axis. Lastly, because most women in the present study were older than 55 years of age, it is uncertain how well the findings herein may generalize to younger study subjects [17].

## Conclusions

The present study reports differences in plasma G activity among racial/ethnic categories, which may provide useful explanation for the wide spectrum of health disparities in different population groups. We also replicated the previously reported positive association between G activity and alcohol intake. Although we could not identify the specific glucocorticoid-like compounds that are acting on the GR, we provide useful information on the association between biological activity of GR, racial/ethnic categories and lifestyle/demographic factors. Advanced mass spectrometry-based technology is needed to identify the specific compounds contributing to the observed associations. In addition to the traditional measurements of cortisol as a biomarker of stress, we highlight that the plasma G activity could be utilized in epidemiologic research as an alternative biomarker that can simultaneously provide insight into biological activity of endogenous glucocorticoids and glucocorticoid-like compounds. Using measurements of G activity can be a useful tool aimed at addressing differences in health disparities among different population groups, particularly in populations that are often exposed to more stressors and experience worse disease outcomes.

## Supporting information

S1 Table Association of sociodemographic and lifestyle factors with plasma glucocorticogenic activity in non-Latina Blacks (N = 99) in the San Francisco Bay Area Breast Cancer Study 1996–2002. (DOCX)

S2 Table Association of sociodemographic and lifestyle factors with plasma glucocorticogenic activity in non-Latina Whites (N = 72) in the San Francisco Bay Area Breast Cancer Study 1996–2002. (DOCX)

S1 Dataset Covariate information used in this study. (XLS)
