# Supplementary material for: Plasma glucocorticogenic activity, race/ethnicity and alcohol intake among San Francisco Bay Area women
Source: PLoS One. 2020 Jun 1;15(6):e0233904. doi: 10.1371/journal.pone.0233904 (PMC7263601; doi:10.1371/journal.pone.0233904)
Supplement: S1 Table — (DOCX) [file pone.0233904.s001.docx]

**S1 Table.** **Association of sociodemographic and lifestyle factors with plasma glucocorticogenic activity in non-Latina Blacks (N=99) in the San Francisco Bay Area Breast Cancer Study 1996-2002.**

| **Characteristics** | **Coefficient (95% CI)** |
| --- | --- |
| Age (yrs) |  |
| <55 | Ref. |
| 55-65 | 0.01 (-0.17, 0.18) |
| >65 | -0.05 (-0.22, 0.13) |
| Height, per 10 cm | -0.13 (-0.25, -0.01) |
| BMI (kg/m^2^) |  |
| <25 | Ref. |
| 25 to <30 | 0.01 (-0.22, 0.24) |
| ≥30 | -0.02 (-0.22, 0.17) |
| Socioeconomic Status (SES) |  |
| 1 (low SES) | Ref. |
| 2 | -0.05 (-0.25, 0.14) |
| 3 | -0.01 (-0.22, 0.19) |
| 4 | 0.01 (-0.25, 0.27) |
| 5 (high SES) | -0.16 (-0.46, 0.13) |
| Alcohol intake per day (gms) |  |
| None | Ref. |
| <10 | 0.17 (-0.04, 0.38) |
| ≥10 | 0.21 (-0.04, 0.47) |

CI, confidence interval.
