# Supplementary material for: Plasma glucocorticogenic activity, race/ethnicity and alcohol intake among San Francisco Bay Area women
Source: PLoS One. 2020 Jun 1;15(6):e0233904. doi: 10.1371/journal.pone.0233904 (PMC7263601; doi:10.1371/journal.pone.0233904)
Supplement: S2 Table — (DOCX) [file pone.0233904.s002.docx]

**S2 Table.** **Association of sociodemographic and lifestyle factors with plasma glucocorticogenic activity in non-Latina Whites (N=72) in the San Francisco Bay Area Breast Cancer Study 1996-2002.**

| **Characteristics** | **Coefficient (95% CI)** |
| --- | --- |
| Age (yrs) |  |
| <55 | Ref. |
| 55-65 | -0.22 (-0.46, 0.03) |
| >65 | -0.09 (-0.30, 0.12) |
| Height, per 10 cm | 0.08 (-0.03, 0.19) |
| BMI (kg/m^2^) |  |
| <25 | Ref. |
| 25 to <30 | -0.03 (-0.22, 0.15) |
| ≥30 | 0.06 (-0.14, 0.26) |
| Socioeconomic Status (SES) |  |
| 1 (low SES) | Ref. |
| 2 | 0.01 (-0.46, 0.48) |
| 3 | -0.13 (-0.56, 0.29) |
| 4 | -0.26 (-0.67, 0.15) |
| 5 (high SES) | -0.33 (-0.72, 0.07) |
| Alcohol intake per day (gms) |  |
| None | Ref. |
| <10 | -0.07 (-0.25, 0.12) |
| ≥10 | 0.05 (-0.14, 0.24) |

CI, confidence interval.
